# Mycothione reductase as a potential target in the fight against *Mycobacterium abscessus* infections

**DOI:** 10.1128/msphere.00669-23

**Published:** 2023-12-12

**Authors:** T. Piller, L. De Vooght, Y. Gansemans, F. Van Nieuwerburgh, P. Cos

**Affiliations:** 1 Department of Pharmaceutical Sciences, Laboratory of Microbiology, Parasitology and Hygiene (LMPH), University of Antwerp, Wilrijk, Belgium; 2 Laboratory of Pharmaceutical Biotechnology, Faculty of Pharmaceutical Sciences, Ghent University, Ghent, Belgium; University of Michigan, Ann Arbor, Michigan, USA

**Keywords:** *Mycobacterium abscessus*, mycothione reductase, knockout, *Galleria mellonella*, ORBIT system, bedaquiline

## Abstract

While infections caused by *Mycobacterium abscessus* complex (MABC) are rising worldwide, the current treatment of these infections is far from ideal due to its numerous shortcomings thereby increasing the urge for novel drug targets. In this study, mycothione reductase (Mtr) was evaluated for its potential as a drug target for MABC infections since it is a key enzyme needed in the recycling of mycothiol, the main low-molecular-weight thiol protecting the bacteria against reactive oxygen species and other reactive intermediates. First, a *Mab*∆*mtr* mutant strain was generated, lacking *mtr* expression. Next, the *in vitro* sensitivity of *Mab*∆*mtr* to oxidative stress and antimycobacterial drugs was determined. Finally, we evaluated the intramacrophage survival and the virulence of *Mab*∆*mtr* in *Galleria mellonella* larvae. *Mab*∆*mtr* demonstrated a 39.5-fold reduction in IC90 when exposed to bedaquiline *in vitro*. Furthermore, the *Mab*∆*mtr* mutant showed a decreased ability to proliferate inside macrophages and larvae, suggesting that Mtr plays an important role during MABC infection. Altogether, these findings support the assumption of Mtr being a potential target for antimycobacterial drugs.

**IMPORTANCE:**

*Mycobacterium abscessus* complex (MABC) is a group of bacteria causing a serious public health problem worldwide due to its ability to cause progressive disease, its highly resistant profile against various antibiotics, and its lengthy treatment. Therefore, new drugs are needed to alleviate antibiotic resistance and reduce the length of the current treatment. A potential new target for new antibiotics is mycothione reductase (Mtr), an important enzyme belonging to a pathway that protects the bacteria against harmful conditions. Our research created a bacterium deficient of *mtr* by using advanced genetic techniques and demonstrated that *mtr*-deficient bacteria have a decreased ability to multiply during infection. Furthermore, we show evidence that currently used antibiotics combined with *mtr* deficiency can lead to a better treatment of MABC infection. Altogether, our results validate Mtr as a potential new target and suggest that Mtr plays a role during MABC infection.

## INTRODUCTION

Infections caused by nontuberculous mycobacteria (NTM) have been increasing worldwide for the past decades**,** leading to a serious public health problem. One of the most clinically relevant species among these NTM is the ones belonging to the *Mycobacterium abscessus* complex (MABC), a group of rapidly growing mycobacteria that are ubiquitous in soil and water (1–3). Around 50% of these MABC infections affect the lungs and are predominantly found in immunocompromised patients with underlying lung disease, such as cystic fibrosis (2, 4, 5). Moreover, MABC pulmonary disease (MABC-PD) in patients with cystic fibrosis has an estimated prevalence of 13% in Europe and 16% in the US (1). Infections caused by this complex are known for being very difficult to treat, as MABC is resistant to the standard antimicrobial agents, its treatment displays a low success rate and often leads to adverse drug effects, and various patients experience relapse after treatment or surgical removal of the pathogen (2, 6, 7). Thus, the current treatment of MABC-PD is far from ideal and requires a long-term multidrug therapy including a macrolide together with two to three intravenous drugs during the initial treatment phase or nebulized amikacin and one to four oral drugs during the continuation phase (2). Accordingly, in order to improve the treatment of MABC infections and relieve the burden of drug resistance, novel mycobacterial targets need to be explored, which effectively reduce the bacterial burden within the host.

An interesting target for the development of anti-mycobacterial drugs is the thiol-based redox homeostasis. This pathway plays a key role in the protection of mycobacteria against harmful endogenous and exogenous reactive oxygen species (ROS) generated by both aerobic respiration and the host’s immune system (8–10). Macrophages, i.e., the primary host cells of MABC during pulmonary disease, will release ROS after phagocytosis to promote bacterial killing (11, 12). The main thiol responsible for the neutralization of ROS in mycobacteria is mycothiol (MSH), a low-molecular-weight thiol found only in actinomycetes (13, 14). MSH is synthesized during a five-step process conducted by a glycosyltransferase (MshA), a phosphatase (MshA2), a deacetylase (MshB), a cysteine ligase (MshC), and an MSH synthase (MshD) (15). Once synthesized, it will act as an antioxidant when exposed to oxidative stress and will subsequently be oxidized into mycothione (MSSM). Then, to recover the reductive intracellular redox environment, MSSM will be reduced back to MSH with the help of an NADPH-dependent enzyme called mycothione reductase (Mtr). Therefore, Mtr plays a major role in the recycling of MSH and hereby maintains a balanced redox homeostasis (10, 16, 17). Recently, several studies have shown that altered levels of MSH affect the susceptibility to oxidative stress and overall survival of other mycobacterial species, including *Mycobacterium tuberculosis* and *Mycobacterium smegmatis* (9, 18).

In this study, we investigated the potential of Mtr as a novel drug target in *Mycobacterium abscessus* (*Mab*). We used a *Mab*∆*mtr* knockout strain to deplete *mtr* expression and showed that although not strictly essential for growth, elimination of *mtr* expression leads to an increased *in vitro* sensitivity toward oxidative stress and antimycobacterial drugs. Furthermore, in the absence of *mtr*, a decrease in intramacrophage replication and survival in *Galleria mellonella* (*G. mellonella*) was observed. Collectively, these results validate Mtr as a potential drug target in *Mab* and demonstrate the potential for synergy by combining currently used antimycobacterial drugs with Mtr inhibitors.

## MATERIALS AND METHODS

### Bacterial strains, media, and culture conditions

All mycobacterial strains in this study were derived from *M. abscessus* ATCC 19977 and were routinely cultured at 37°C in Middlebrook 7H9 broth (Sigma) supplemented with 10% ADS (albumin-dextrose-saline), 0.2% glycerol, and 0.05% tyloxapol or Sauton’s medium (HiMedia Laboratories) supplemented with 2% glycerol and 0.05% tyloxapol with the addition of 100 µg/mL Zeocin (Fisher Scientific) for the *Mab*∆*mtr* mutant. Agar plates were made of Middlebrook 7H11 agar base (Sigma) or Sauton agar, consisting of Sauton’s medium solidified with 1.5% Bacto Agar (Becton, Dickinson and Company).

### Construction of a *Mab*∆*mtr* mutant

For the construction of the *Mab*∆*mtr* mutant, the ORBIT (Oligo Recombineering followed by Bxb1 Integrase) system was used as described by Murphy et al. (19). Briefly, *Mab* was grown in a shaking incubator (New Brunswick Scientific; 175 rpm) in 7H9 supplemented with 10% OADC (oleic acid-albumin-catalase-dextrose; Thermo Fisher Scientific), 0.2% glycerol, and 0.05% tyloxapol at 37°C until reaching an optical density at 600 nm (OD_600_) between 0.2 and 0.8 and was made electrocompetent by washing three times with 10% glycerol. Then, 1 µg of the endogenous PKM444 plasmid (Addgene 108319) was transformed into the electrocompetent bacteria by using the Gene Pusler Xcell Total System (Bio-Rad; 1.25 kV, 1,000 Ω and 25 µF). Following electroporation, *Mab* was resuspended in 7H9 medium containing 20% OADC and incubated at 37°C for 4 h before it was plated out on 7H11 agar containing 10% OADC, 0.2% glycerol, and 200 µg/mL kanamycin (Sigma). The 7H11 agar plates were incubated for 3 days to 1 week at 37°C until the presence of colonies. After confirming plasmid uptake by the candidate colonies by PCR using primers amplifying the kanamycin-resistant cassette of the PKM444 plasmid, *Mab*::PKM444 was grown in the same medium, induced for plasmid expression with 500 ng/mL anhydrotetracycline (Takara Bio Europe) 18 h before electroporation and incubated in the shaking incubator at 37°C until reaching the previously mentioned OD_600_. Next, the induced culture was made electrocompetent and transformed together with 1 µg of the *attP*-containing oligonucleotide and 200 ng of the *attB*-containing PKM496 plasmid (Addgene 109301) using the same system and settings as the previous transformation. The transformed bacteria were incubated in the shaking incubator in the 7H9 medium containing 20% OADC at 37°C overnight to allow for homologous recombination before being spread out on 7H11 agar plates supplemented with 10% OADC, 0.2% glycerol, and 100 µg/mL Zeocin and incubated for 1–2 weeks at 37°C. Colonies obtained from the transformation were confirmed for successful recombination and thus successfully obtained *Mab*∆*mtr* by PCR and Sanger sequencing (Neuromics Support Facility, University of Antwerp) utilizing primers to amplify the wild-type (WT) *mtr* gene together with the fully incorporated PKM496 plasmid into the WT *mtr* (Table S1). All primers and oligos used in this study are listed in Table S1.

### Whole-genome sequencing and analysis

Genomic DNA concentration was determined using Quant-iT PicogGreen dsDNA (Thermo Fisher) and integrity was inspected on a 1% E-gel (Invitrogen). About 120–800 ng input DNA was fragmented in a Covaris S2 sonicator, aiming for 400-bp fragments. For each sample, a sequencing library was constructed using the NEBNext Ultra II DNA Library Prep Kit for Illumina (New England Biolabs) using 90–400 ng of fragmented material. After adapter ligation, library fragments were size-selected for 400–800 bp on a 2% E-gel and purified using the Zymoclean Gel DNA Recovery Kit (Zymo Research). Half of the material was then submitted to six PCR cycles and purified using Ampure XP beads (Beckman Coulter). Quality was checked using a High Sensitivity DNA Kit on a Bioanalyzer (Agilent). Yield was determined by qPCR according to the “Sequencing Library qPCR Quantification Guide” (Illumina). Sample libraries were pooled equimolar and a final size selection for 400–800-bp fragments was done on a 2% E-gel. The material was purified using the Zymoclean Gel DNA Recovery Kit. The pooled libraries were sequenced as paired-end 150 on a NovaSeq device (Illumina).

Sequencing read quantity and quality were evaluated using FastQC (v0.11.9) (20). Contamination was checked using FastQ Screen (v0.15.1) (21) and genomes from a limited set of common lab organisms. Adapter trimming and quality trimming were done with cutadapt (v3.7) (22) using a phred score threshold of 20 and removing reads with ambiguous bases. We used breseq (v0.37.0) (23) to perform structural variant analysis using either the *Mab L948* (ATCC 19977) reference genome (GCF_000069185.1_ASM6918v1_genomic.gbff GenBank file from NCBI) or the *Mycobacterium tuberculosis H37Ra* (ATCC 25177) reference genome (GCF_001938725.1_ASM193872v1_genomic.gbff GenBank file from NCBI), together with plasmid sequences and putative genome-inserted sequences. Briefly, the tool first maps the trimmed reads on the reference sequences with bowtie2 (v2.4.5) (24), then performs a variant analysis and reports SNPs, as well as new junctions explaining larger deletions and insertions. Finally, it annotates all detected mutations using the available genome information, and the results are reported as an interactive HTML document.

### RNA isolation

*Mab* strains were grown in 7H9 supplemented with 10% ADS, 0.2% glycerol, and 0.05% tyloxapol until reaching their logarithmic phase and diluted to an OD_600_ of 0.1 in the same medium. After 48 h of growth in a shaking incubator (New Brunswick Scientific; 175 rpm) at 37°C, the pellets of the strains were harvested and incubated in TRIzol reagent (Invitrogen) for 5 min at room temperature (RT). Next, the bacteria were lysed with BeadBug beads (Sigma; 0.1 mm Zirconium beads) by shaking at a speed of 6 m/second twice for 45 seconds using the FastPrep 24 Classic (MP biomedicals) followed by overnight incubation at −80°C. In order to separate the samples from the TRIzol reagent, Phasemaker tubes (Invitrogen) were used together with the addition of chloroform to the sample. Once separated, RNA isolation of the samples was completed using the RNeasy Plus Mini Kit (Qiagen) followed by a DNase treatment completed with TURBO DNAase (Qiagen) and ezDNase (Invitrogen). The final RNA concentration was measured using the NanoDrop 2000 spectrophotometer (Thermo Scientific), and the RNA samples were stored at −80°C until further use.

### Real-time quantitative PCR

All real-time quantitative PCRs (RT-qPCRs) were performed combining 10  µL of 2× SensiFAST SYBR No-ROX One-Step mix (Biotech), 0.6  µL of each primer (0.3  µM final concentration; Table S1), 0.2  µL of reverse transcriptase (Biotech), 0.4  µL of RNase inhibitor (Biotech), 3  µL of RNA template, and 5.2  µL of diethylpyrocarbonate-treated water (Biotech) in each well to reach a final volume of 20 µL. Next, the mRNA expression was measured using LightCycler 480 system (Roche) with predetermined cycle conditions [reverse T1 (45°C, 10 min, 1×), two-step amplification (95°C, 5 seconds; 60°C, 30 seconds, single, 40×), and melting (95°C, 10 seconds; 45°C, 1 min; 95°C, continuous, 1×)] and analyzed relative to the expression of the housekeeping gene, *rpoB*, with the LightCycler 480 SW 1.5.1 software. The normalized relative expression levels were further analyzed using GraphPad software 8.0. All primers are listed in Table S1.

### Quantification of intracellular reduced thiol levels

For the quantification of the intracellular reduced thiol levels, the Thiol Fluorescent Detection Kit (Thermo Fisher Scientific) was used. Briefly, mycobacterial strains were grown in 7H9 broth supplemented with 10% ADS, 0.2% glycerol, and 0.05% tyloxapol until reaching their logarithmic phase, diluted in the same medium to match an OD_600_ of 0.1 and incubated at 37°C for 48 h. After 48 h, the cultures were washed twice with DPBS (Dulbecco’s phosphate-buffered saline; Gibco) supplemented with 0.05% tyloxapol and resuspended in 1× assay buffer (Thermo Fisher Scientific). Next, the mycobacterial cell wall was disrupted with BeadBug beads (Sigma; 0.1 mm zirconium beads) by shaking at a speed of 6 m/second twice for 60 seconds using the FastPrep 24 Classic (MP biomedicals) followed by centrifugation of the cultures and isolation of the supernatants. The samples were diluted in a one-over-two manner by using the 1× assay buffer after which 100 µL of each diluted sample was added to a black half area 96-well plate together with 25 µL detection reagent (Thermo Fisher Scientific). The plate was incubated for 30 min at RT in the dark before reading the fluorescent signal with the Tecan plate reader (Infinite F plex) at an emission of 510 nm and excitation of 390 nm. For the determination of the thiol levels, the fluorescent values of the samples were plotted according to a standard curve obtained with an N-Acetylcysteine standard (Thermo Fisher Scientific).

### Growth curves

The bacteria were grown until the logarithmic phase in 7H9 broth supplemented with 10% ADS, 0.2% glycerol, and 0.05% tyloxapol or Sauton’s medium supplemented with 2% glycerol and 0.05% tyloxapol and diluted to an OD_600_ of 0.05 before being incubated in a shaking incubator (New Brunswick Scientific; 175 rpm) at 37°C. Growth of the strains was evaluated every 24 h by measuring the OD_600_ with a cell density meter (Biochrom WPA Biowave). In parallel, the same experiment was conducted for 2 days in 7H9 broth and 3 days in Sauton’s medium with growth measured by both OD_600_ and CFU count to determine the CFU-OD_600_ proportion of each strain.

### Oxidative stress assay

Logarithmic-phase mycobacterial strains were cultured in Sauton’s medium supplemented with 2% glycerol and 0.05% tyloxapol after which they were diluted to an OD_600_ of 0.05. At that moment, all strains were divided into two groups, whereas one group was subjected to 15 mM hydrogen peroxide (H_2_O_2_) before they were all incubated in a shaking incubator (New Brunswick Scientific; 175 rpm) at 37°C. A part of the cultures was harvested at 0, 4, 8, and 24 h after the addition of H_2_O_2_. The ATP levels of the cultures were measured using the BacTiter-Glo kit (Promega), and a 10-fold serial dilution of each culture was plated on 7H11 agar plates supplemented with 10% ADS and 0.2% glycerol for measurement by CFU count.

### Antimicrobial activity determination

The selected compounds, bedaquiline (Sigma), clofazimine (Sigma), and moxifloxacin (Sigma), were first solubilized in 100% dimethyl sulfoxide (DMSO; Sigma) at a concentration of 20 mM and stored at −20°C until further use. To determine the activity of the compounds against the mycobacterial strains, both *Mab* WT and *Mab*∆*mtr* mutants in their logarithmic phase were diluted to an OD_600_ of 0.05 with 7H9 supplemented with 10% ADS, 0.2% glycerol, and 0.5% tyloxapol and inoculated in a 96-well plate. Then, each compound was added in a one-over-three dilution to the 96-well plates containing the bacteria to reach a final concentration starting from 100 µM and a maximal final DMSO concentration of 1%. The 96-well plates were incubated at 37°C for 3 days to allow for exposure to the compounds. After incubation, 0.001% (wt/vol) of resazurin (Sigma) was added to each well after which the plates were incubated again overnight at 37°C. Finally, the viability of the mycobacterial strains was assessed by measuring the fluorescence signal emitted by each well at an excitation and emission of 550 and 590 nm, respectively, with the use of a Tecan plate reader (Infinite F plex).

### RAW 264.7 macrophage infection

RAW 264.7 murine macrophages were cultured in Dulbecco’s modified Eagle’s medium (DMEM; Thermo Fisher Scientific) containing 10% heat-inactivated fetal calf serum (iFCS; Thermo Fisher Scientific), 10% Penicillin-Streptomycin (Thermo Fisher Scientific; 10,000 U/mL), and 10% L-Glutamine (Glutamax; Thermo Fisher Scientific; 200 nM) at 37°C. To determine the infectivity of the different mycobacterial strains, the macrophages were seeded in a 24-well plate (Greiner Bio-One) in DMEM supplemented with 5% iFCS at a concentration of 5 × 10^5^ cells/mL and incubated overnight at 37°C. Next, the cells were infected at a multiplicity of infection (MOI) of 5 during 4 h at 37°C in the presence of 5% CO_2_. After infection, 200 µg/mL amikacin (Sigma) was added to the cells, and they were incubated once more in the same conditions for 45 min to kill all extracellular bacteria. The bacterial load was analyzed 0 and 24 h after infection by first lysing the cells with 0.1% Triton-X-100 for 10 min and then plating out a serial dilution on 7H11 agar plates supplemented with 10% ADS and 0.2% glycerol to determine the CFUs.

### *Galleria mellonella* infection

*G. mellonella* larvae were purchased from Anaconda Reptiles (Kontich, Belgium) and stored in boxes filled with wood chips at 4°C. The protocol followed was adapted from Cools et al. (25) and Meir et al. (26). For infection, larvae were injected in the penultimate pro-leg with 5 × 10^3^ CFU in a volume of 10 µL by a 31G needle using a Hamilton syringe. At the same time, the control group was injected with 10 µL DPBS (Gibco). Next, the larvae were incubated at 37°C until they were sacrificed or until the end of the experiment. To generate a Kaplan-Meier curve, a total of 90 larvae received a dead-or-alive score every 24 h based on the absence of movement in response to external stimuli and melanization of the larvae. For CFU count of the bacteria per larvae, a total of eight to eleven larvae of each infected group were sacrificed by freezing for 30 min on days 2, 4, and 6. Then, these larvae were decontaminated with 70% ethanol, homogenized by the Qiagen TissueRuptor and plated out in a serial dilution on 7H11 agar containing 10% ADS, 0.2% glycerol, 2 µg/mL vancomycin (Sigma), and 8 µg/mL ceftazidime (Sigma). The plates were incubated at 37°C until the colonies could be counted properly.

### Statistical analysis

The Mann-Whitney test was applied to statistically evaluate the results obtained by RT-qPCR, oxidative stress assay, macrophage assay, and *G. mellonella* infection, and it was followed by correction for multiple testing when required. The interpretation of a decline or increase of the results over time was statistically analyzed with a non-linear regression, while the difference in the obtained results over time during *G. mellonella* infection was analyzed using the Kruskal-Wallis test. The Kaplan-Meyer curve was analyzed using the Log-rank (Mantel-Cox) test. The results were considered significantly different when *P* < 0.05. All statistical analysis was performed using the Graphpad software 8.0.

## RESULTS

### Part of the MSH biosynthesis pathway is upregulated when its recycling pathway is disabled

**Fig 1:**
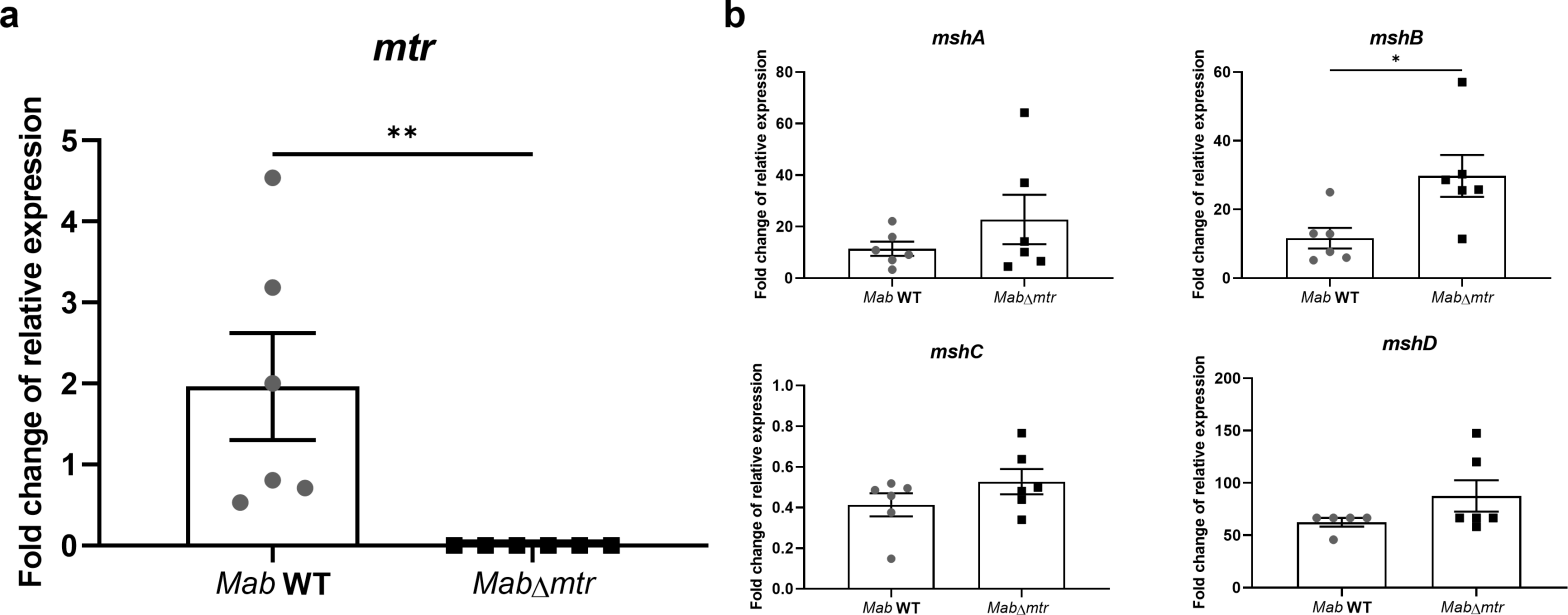
Part of the MSH biosynthesis pathway is upregulated in the confirmed *Mab*∆*mtr* mutant. Log-phase bacteria were diluted to an OD_600_ of 0.01 and incubated for 48 h at 37°C before RNA was isolated. The RNA samples were examined by RT-qPCR after which mRNA expression of *mtr*, *mshA*, *mshB*, *mshC,* and *mshD* was analyzed relative to the expression of the *rpoB* gene. (a) Absence of *mtr* expression in the *Mab*∆*mtr* confirms successful development of an *mtr* knockout. (b) Analysis of the mRNA expression of the MSH biosynthesis pathway genes *mshA*, *mshB*, *mshC,* and *mshD*. A significant difference in relative expression between the WT and *Mab*∆*mtr* is observed for the *mshB* gene alone meaning only part of the MSH biosynthesis pathway is upregulated when *mtr* is disabled. Results are shown as mean ± SEM from six independent experiments. Statistical significance was obtained with the Mann-Whitney test. *P  <  0.05 and **P < 0.01.

After establishing the correct implementation of the ORBIT system to produce a *Mab*∆*mtr* mutant (Fig. S1), the WT and *Mab*∆*mtr* strains were analyzed by whole-genome sequencing (WGS) to exclude the occurrence of off-target effects during transformation (Table S2). The WGS results confirmed that no off-target integration of the oligo and PKM496 plasmid occurred in the *Mab*∆*mtr* mutant. Next, to confirm the successful knocking out of the *mtr* gene in the *Mab*∆*mtr* mutant, first, the relative expression of *mtr* was evaluated in the WT and *Mab*∆*mtr* using RT-qPCR (Fig. 1a). As observed in the figure, the *Mab*∆*mtr* mutant displays no expression of *mtr*, hereby confirming the abolishment of *mtr* expression after deletion of the gene. To evaluate compensation for the loss of *mtr*, i.e., the MSH recycling pathway, the relative expression of *mshA*, *mshB*, *mshC*, and *mshD* was analyzed as well. No significant difference was detected between the strains for the *mshA*, *mshC,* and *mshD* gene expression (Fig. 1b). Interestingly, the relative expression of *mshB* showed a 2.6-fold upregulation in *Mab*∆*mtr* compared to the WT indicating that only part of the MSH biosynthesis pathway is upregulated when the recycling pathway is not expressed.

### *Mab*∆*mtr* shows a decrease in the intracellular reduced thiol levels

**Fig 2:**
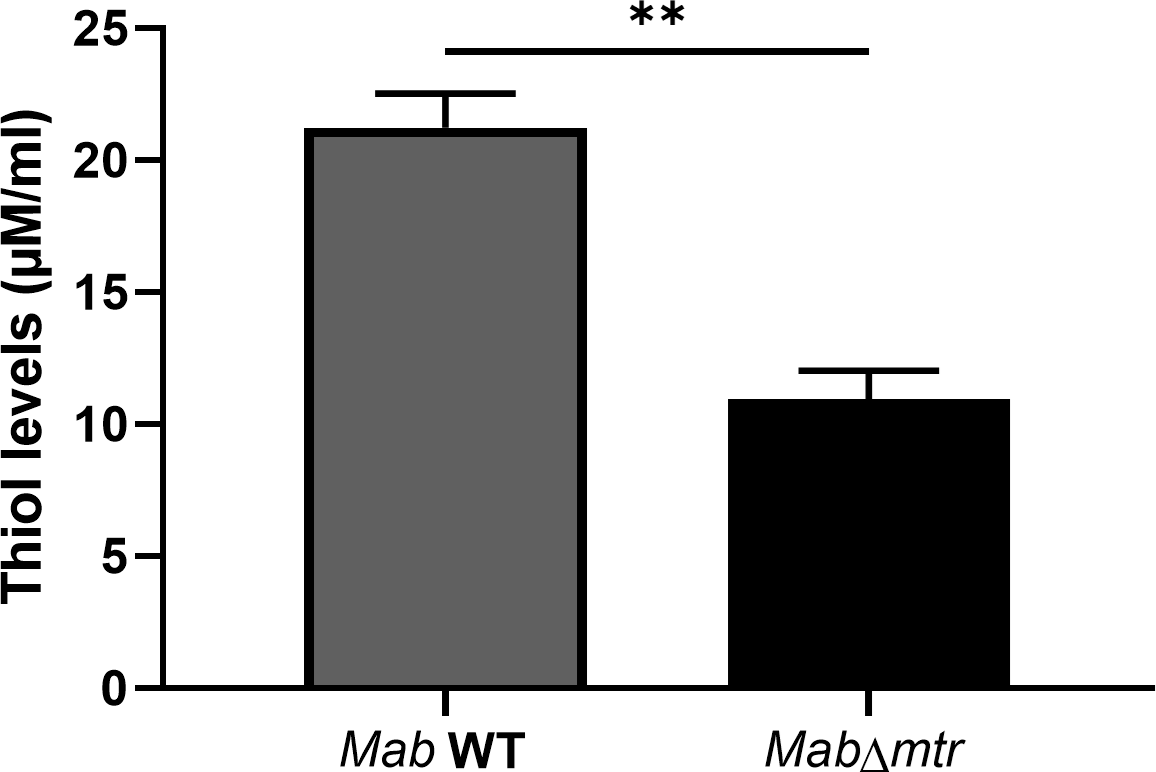
Intracellular reduced thiol levels are diminished in *Mab*∆*mtr* mutant. The total intracellular reduced thiol levels were measured in the lysed *Mab* WT and *Mab*∆*mtr* strains after growing for 48 h. WT *Mab* cultures presented intracellular thiol concentrations of around 21 µM/ml. Additionally, a 1.9-fold decrease in intracellular thiols is detected after knocking out *mtr*. Results are shown as mean ± SEM from three independent experiments. Statistical significance was obtained with the Mann-Whitney test. **P < 0.01.

To investigate whether knocking out *mtr* translates into a decrease in thiol levels, the intracellular reduced thiol levels were measured after lysis of the mycobacterial cell wall of cultures grown for 48 h. It was observed in Fig. 2 that the WT culture contains a total concentration of intracellular thiols of around 21 µM/ml. Moreover, *Mab* displays a significant decrease of 1.9-fold in intracellular thiols after *mtr* is disabled.

### Knocking out *mtr* enables *Mab* to reach a higher plateau phase in a nutrient-poor medium

**Fig 3:**
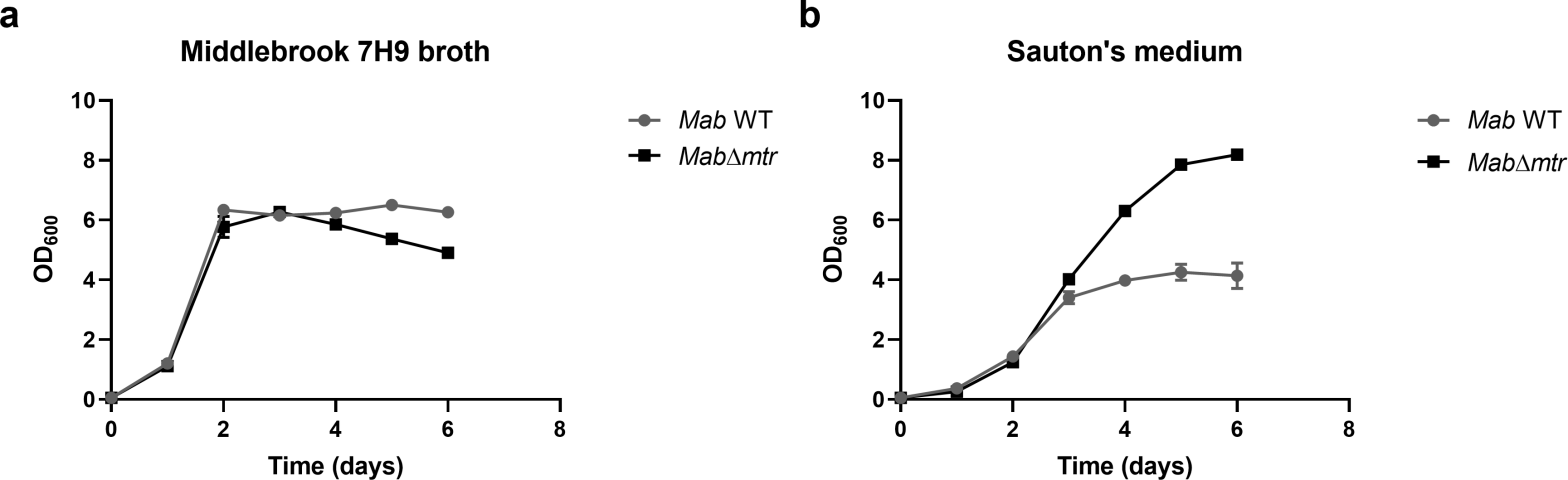
*Mab*∆*mtr* mutant reaches a higher plateau phase than *Mab* WT in a nutrient-poor medium. Growth curve of the WT and *Mab*∆*mtr* mutant in Middlebrook 7H9 broth supplemented with 10% ADS, 0.2% glycerol, and 0.05% tyloxapol (**a**) and nutrient-poor Sauton’s medium supplemented with 2% glycerol and 0.05% tyloxapol (**b**) shaking at 37°C. Before incubation, both strains were grown until their logarithmic phase and diluted in the corresponding medium to reach an OD_600_ of 0.05. (**a**) Both the WT and *Mab*∆*mtr* mutant strains grew in the same way in a nutrient-rich medium regardless of the presence or absence of Mtr. (**b**) Remarkably, a significant difference in growth is observed between the WT and *Mab*∆*mtr* when growing in the nutrient-poor Sauton’s medium. Hereby, *Mab*∆*mtr* reached a higher plateau than the WT. Results are shown as mean ± SEM from three independent experiments. A non-linear regression for Gompertz growth with the least square fit was used to analyze the curves.

Since *Mab* can survive nutrient starvation for extended periods of time, bacterial growth of the WT and *Mab*∆*mtr* strains was assessed in a nutrient-rich and nutrient-poor medium, Middlebrook 7H9 broth, and Sauton’s medium, respectively. The strains were incubated starting from an OD_600_ of 0.05 while shaking at 37°C and evaluated every 24 h for their growth based on OD_600_. When growing in a nutrient-rich medium, the absence of *mtr* expression had no effect on the growth of both strains (Fig. 3a). Surprisingly, a significant difference is observed in growth between WT *Mab* and *Mab*∆*mtr* when growing in Sauton’s medium with *Mab*∆*mtr* reaching a higher plateau phase (Fig. 3b). These observations were confirmed for both strains by CFU count while establishing that the mutation in *Mab*∆*mtr* did not alter the CFU-OD_600_ proportion of *Mab* in a nutrient-rich medium (Fig. S2).

### *Mab*∆*mtr* demonstrates a fast reduction of ATP levels under oxidative stress conditions

**Fig 4:**
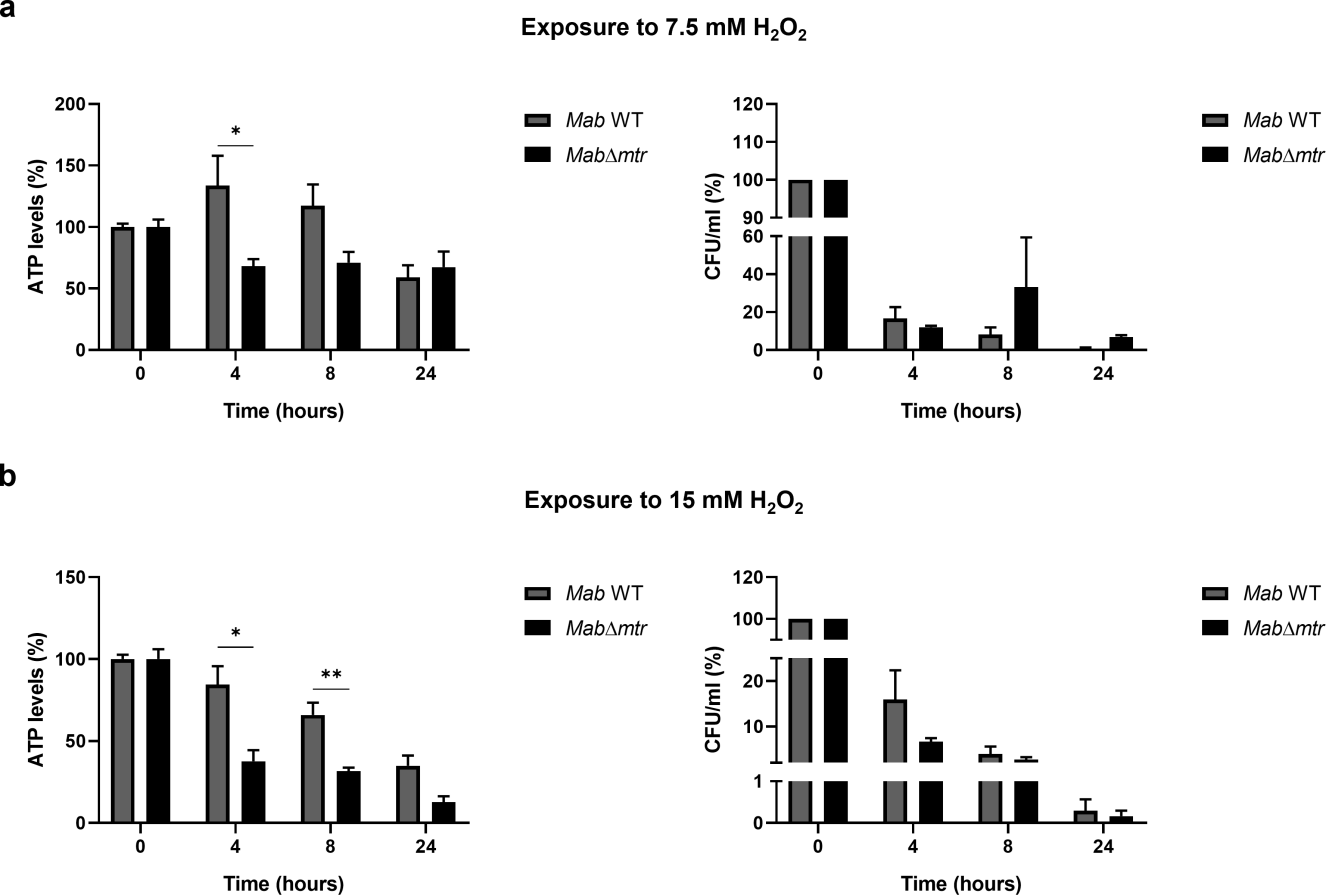
A fast reduction of ATP levels is observed in *Mab*∆*mtr* after exposure to oxidative stress. As Mtr plays a key role in the protection mechanism of *Mab* against oxidative stress, both WT and *Mab*∆*mtr* were exposed to 7.5 or 15 mM H_2_O_2_. After 0, 4, 8, and 24 h of exposure, the ATP levels and viability of the strains were assessed. (**a**) After the addition of 7.5 mM H_2_O_2_ to both strains, the ATP levels of *Mab*∆*mtr* were significantly lower after 4 h of exposure but did not translate into a change in CFU. (**b**) A significant difference in ATP levels is observed between both strains after 4 and 8 h of being cultured in 15 mM H_2_O_2_. Furthermore, the ATP levels of *Mab*∆*mtr* declined faster over time than the WT, showing a reduced metabolic active *Mab* when *mtr* is disabled. However, a greater reduction in ATP levels did not lead to a change in viability between the WT and *Mab*∆*mtr* after exposure to H_2_O_2_. Results are shown as mean ± SEM from three independent experiments after normalization of the results collected after the addition of 15 mM H_2_O_2_ relative to the results acquired when no H_2_O_2_ was added to the cultures. Hereby, the cultures to which no H_2_O_2_ was added were displayed as 100% to enable the results to demonstrate the extent of change in ATP levels or CFU in percentage after the addition of H_2_O_2_ to each strain. Statistical significance was obtained with the Mann-Whitney test followed by correction for multiple testing. To analyze the decay of ATP or CFU/mL for each strain, a non-linear regression for a one-phase decay with least square fit was used. *P  <  0.05 and **P < 0.01.

When infecting a host, *Mab* is subjected to various types of endogenous and exogenous oxidative stress, including hydrogen peroxide. Given the importance of Mtr in neutralizing this oxidative stress, both *Mab* WT and *Mab*∆*mtr* strains were analyzed for their sensitivity against H_2_O_2_ in the nutrient-poor Sauton’s media. For this experiment, logarithmic-phase bacterial cultures were diluted to an OD_600_ of 0.05, followed by the addition of 7.5 or 15 mM H_2_O_2_ to half of each culture and incubation while shaking at 37°C. After 0, 4, 8, and 24 h of exposure, the ATP levels of the cultures were determined with the BacTiter-Glo kit as well as the viability of the strains by CFU count. It is observed in Fig. 4a that *Mab*∆*mtr* demonstrated lower ATP levels than the WT after 4 h of exposure to 7.5 mM H_2_O_2_. However, this reduction in ATP levels did not affect the CFU. After exposure to 15 mM H_2_O_2_, the ATP levels of the mutant strain were lower than that of the WT after 4 and 8 h exposure to 15 mM H_2_O_2_. Furthermore, a faster decay of ATP levels was perceived over time in *Mab*∆*mtr* compared to the WT after H_2_O_2_ was added, corresponding to a reduction in metabolic activity. This lower metabolic state of the *Mab*∆*mtr* mutant strain had no effect on the viability of the strain (Fig. 4b).

### The absence of *mtr* causes *Mab* to be more susceptible to bedaquiline

To further evaluate whether *Mab*∆*mtr* is more susceptible to oxidative stress and reduced ATP levels, the strains were subjected to antimycobacterial compounds known to target the ATP synthase (bedaquiline), generate ROS (clofazimine), or target DNA replication (moxifloxacin) (27). For this purpose, logarithmic-phase *Mab* WT and *Mab*∆*mtr* were incubated at an OD_600_ of 0.05 after which the compounds were added in a one-over-three dilution. Out of all compounds, bedaquiline generated the greatest shift in the susceptibility of the strains with *Mab*∆*mtr* displaying a 5.1-fold reduction in IC50 and a 39.5-fold reduction in IC90 compared to the WT (Table 1). Moreover, a 2.4-fold decline of the IC90 is observed in the *Mab*∆*mtr* mutant when treated with moxifloxacin. Curiously, the activity of clofazimine remains unaltered after disabling *mtr*.

**TABLE 1:** A higher susceptibility to bedaquiline is obtained when *Mab* lacks *mtr^a^*

Drug	*MabWT*		*MabΔmtr*	
	EC50 (µM)	EC90 (µM)	EC50 (µM)	EC90 (µM)
Bedaquiline	0.51± 0.33	9.08 ± 3.16	0.10 ± 0.05	0.23 ± 0.10
Clofazimine	4.36 ± 0.34	6.91 ± 1.75	3.28 ± 0.74	7.97 ± 0.80
Moxifloxacin	1.74 ± 0.36	4.97 ± 1.28	1.14 ± 0.21	2.05 ± 0.63

^*a*^ A panel of three antimycobacterial drugs was selected targeting the ATP-synthase, generating oxidative stress, or targeting the DNA replication; bedaquiline, clofazimine, and moxifloxacin, respectively. The compounds were added in a one-over-three dilution to WT and *Mab*∆*mtr* cultures set at an OD_600_ of 0.05. The results show a considerable shift in the susceptibility of *Mab*∆*mtr* compared to *Mab* WT when subjected to bedaquiline, demonstrating a 5.1-fold reduction in IC50 and a 39.5-fold reduction in IC90. Moxifloxacin demonstrated a higher activity against *Mab*∆*mtr* as well with a 2.4-fold decrease in IC90. However, no difference in activity was detected when clofazimine was added to the strains, further confirming that oxidative stress does not lead to a reduction in the viability of *Mab*∆*mtr*. Results are expressed as the average of three individual experiments ± SD with SD = $\sqrt{\frac{\sum {\left(X_{i}-\overset{-}{X}\right)}^{2}}{n-1}}$ . The IC50 and IC90 of each individual experiment were calculated using GraphPad software 8.0.

### *Mab*∆*mtr* lacks the ability to proliferate inside macrophages

**Fig 5:**
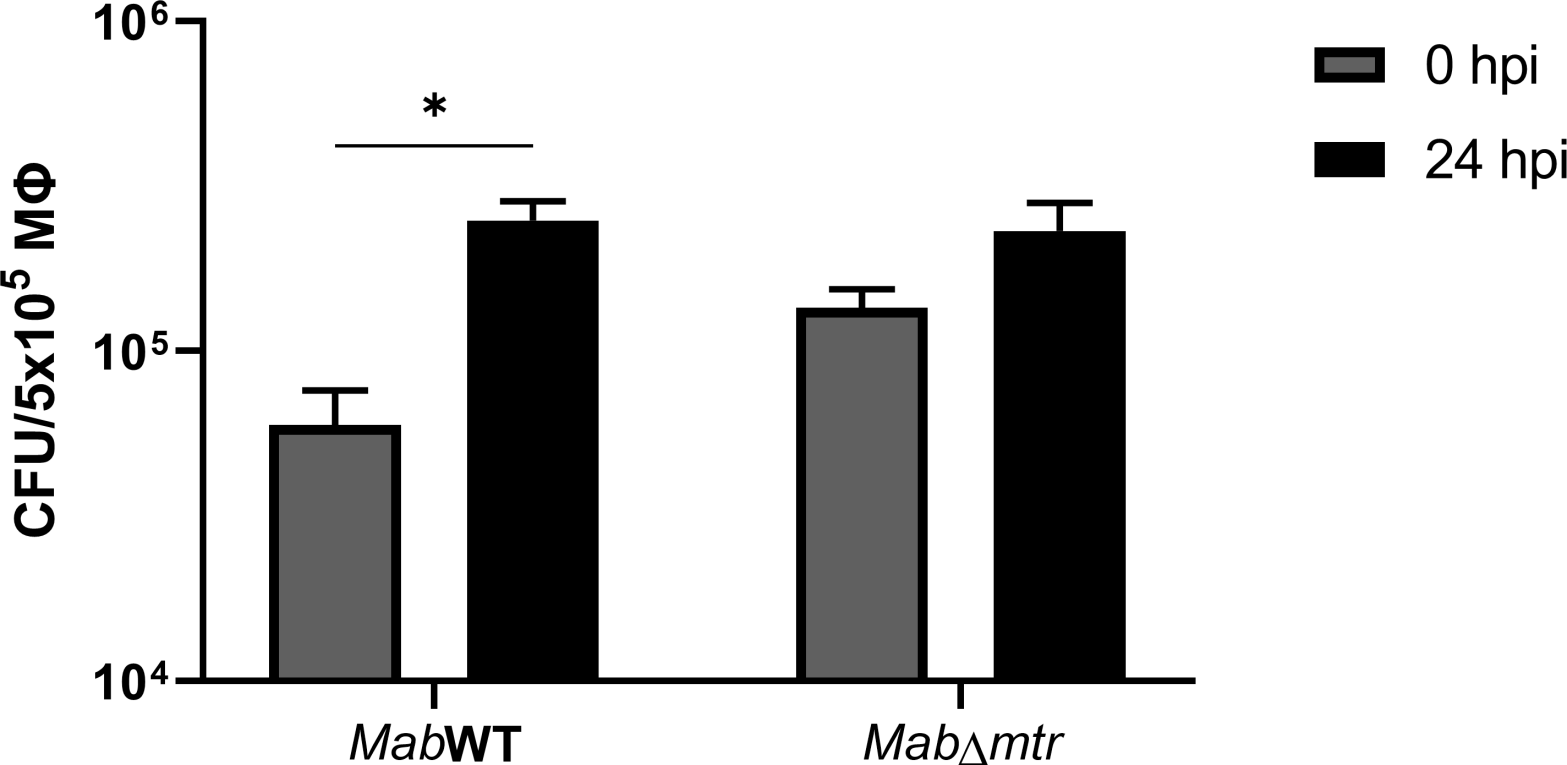
No proliferation of *Mab*∆*mtr* is observed intracellularly. RAW 264.7 macrophages were infected with *Mab* WT and *Mab*∆*mtr* at an MOI of 5 and were lysed at 0 and 24 h post-infection (hpi) to determine the intracellular survival and replication. At 0 hpi, no difference was observed in the intracellular CFU of macrophages infected with either *Mab* WT or *Mab*∆*mtr*. The intracellular replication of the WT strain showed a significant increase after 24 h, while *Mab*∆*mtr* failed to proliferate. Results are shown as mean ± SEM from three independent experiments, and each independent experiment was performed in double. Statistical significance was obtained with the Mann-Whitney test followed by correction for multiple testing. *P  <  0.05.

Macrophages are natural host cells of *Mab* during infection (12). Therefore, the intracellular proliferation of both strains was characterized by setting up an *in vitro* macrophage assay in which RAW 264.7 macrophages were infected with an MOI of 5 with either *Mab* WT or *Mab*∆*mtr*. After infection, part of the macrophages was lysed directly to evaluate the actual infection, while the remaining macrophages were lysed after 24 h to determine the extent of intracellular replication. As displayed in Fig. 5, a significant increase in proliferation of the WT inside macrophages was detected over time, while *Mab*∆*mtr* was not able to proliferate inside the macrophages after 24 h. No difference was observed in the initial infection with the WT or *Mab*∆*mtr*.

### Knocking out *mtr* inhibits the proliferation of *Mab* inside *G. mellonella* larvae

**Fig 6:**
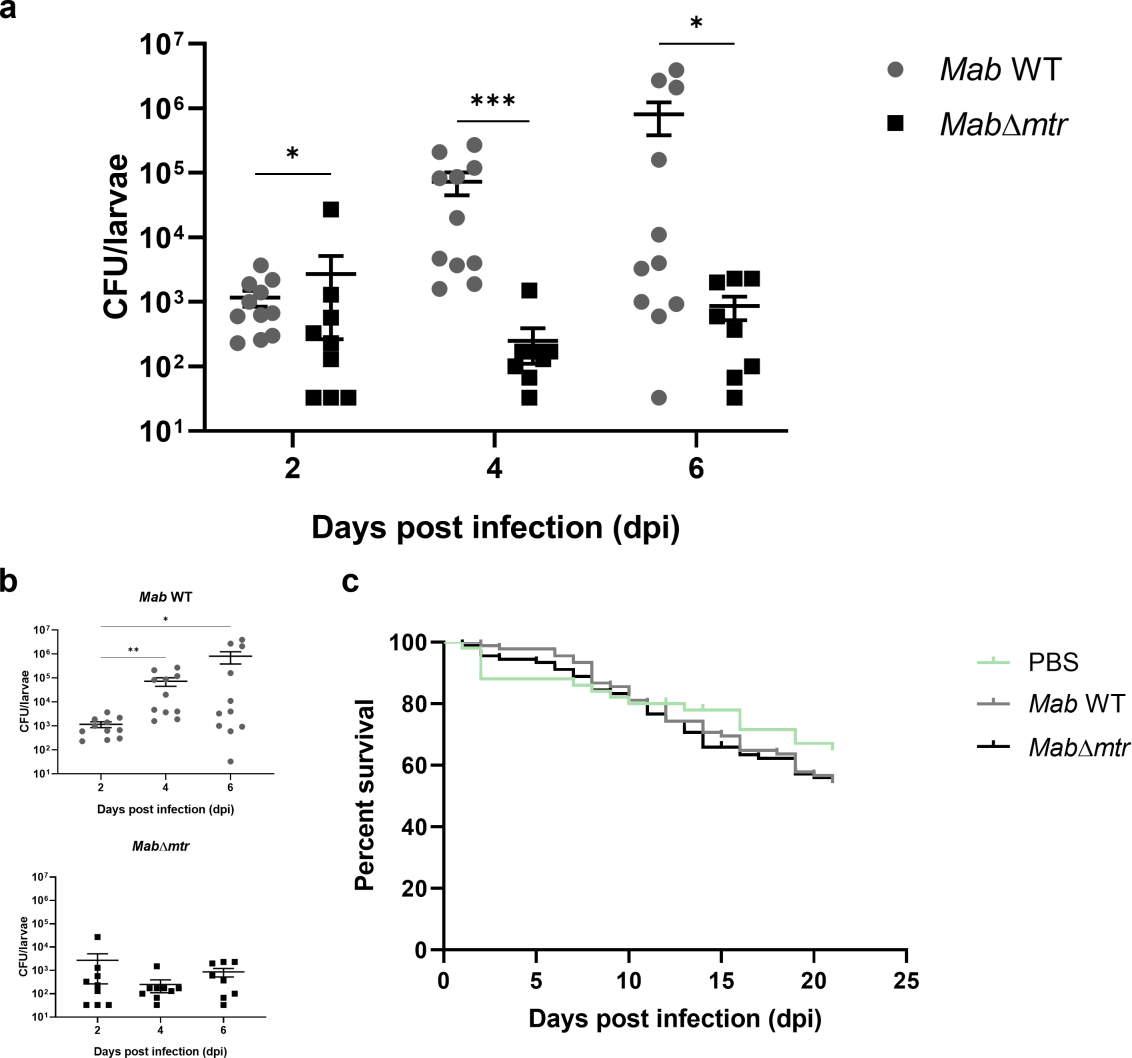
*Mab*∆*mtr* has a reduced ability to proliferate inside *G. mellonella* larvae. *Mab* WT and *Mab*∆*mtr* were used to infect *G. mellonella* larvae with 5 × 10^3^ bacteria per larva. The larvae were incubated at 37°C until 8–10 larvae per condition were sacrificed at 2, 4, and 6 days after infection, and the CFU count was assessed to ascertain the internal bacterial load (**a and b**). In parallel, 90 larvae were infected with each strain to investigate whether the different strains influence the survival of the larvae (**c**). Fifty larvae were injected with PBS as a control. Survival of the larvae was determined by a dead-or-alive score every 24 h. (**a**) At each timepoint after infection, the CFU count of the WT was significantly higher than that of the *Mab*∆*mtr* mutant. (**b**) Proliferation of the WT inside the larvae increased over time, whereas the *Mab*∆*mtr* was unable to proliferate inside the larvae. (**c**) A higher bacterial load within the larvae did not lead to a reduced survival of WT-infected larvae. Moreover, no difference in survival is observed between the groups infected with both bacteria and the control group. Results are shown as mean ± SEM from two independent experiments. Statistical significance was obtained with the Mann-Whitney test followed by correction for multiple testing or by performing a survival analysis with the Log-rank (Mantel-Cox) test. *P  <  0.05 and ***P < 0.001.

To further unravel the role of Mtr during *Mab* infection, *G. mellonella* larvae were used as an *in vivo* infection model for *Mab*. Each larva was infected with 5 × 10^3^ bacteria with either WT or *Mab*∆*mtr* and kept at 37°C. Eight to ten larvae per condition were sacrificed on days 2, 4, and 6 after infection to assess the bacterial load in each larva by CFU count. In parallel, a total of 90 larvae injected with each strain and an additional 50 larvae injected with PBS received a dead-or-alive score every 24 h to define whether infection with the different strains affects the survival of the larvae. The results indicate that larvae infected with the WT showed a significantly higher CFU count compared to the *Mab*∆*mtr* mutant strain at each timepoint after infection (Fig. 6a). Furthermore, while WT *Mab* was able to proliferate inside the larvae over time, proliferation of *Mab*∆*mtr* over time was absent (Fig. 6b). However, the differential ability of both strains to proliferate within the larvae did not affect the survival of the larvae (Fig. 6c). Neither was a difference observed in the survival between the larvae infected with the WT, *Mab*∆*mtr* mutant, and PBS.

## DISCUSSION

MABC outbreaks and nosocomial transmissions are rising worldwide, leading to a serious public health problem (5). Unfortunately, the current treatment of MABC infections is very limited due to the extensive drug-resistant profile of this pathogen. As a result, treating these infections requires a lengthy and complex treatment, which is frequently characterized by high failure rates, serious adverse drug effects, and acquired drug resistance (7, 28, 29). Hence, there is an urgent need for novel mycobacterial drug targets and treatment options.

Mycobacteria are constantly exposed to endogenous and exogenous oxidative stress stimuli when infecting a host (8–10). They are described to be highly sensitive to oxidative stress (30, 31) but are able to neutralize most of it inside a host by means of MSH and other pathways (18). Several studies have already highlighted the importance of MSH together with its biosynthesis and recycling pathway and presented them as potential drug targets in *M. tuberculosis, M. smegmatis*, and *Mycobacterium intracellulare* (15, 31–33). Coulson et al. demonstrated that the growth of *M. tuberculosis* is inhibited when the MSH biosynthesis and MSH-dependent detoxification are lost (30). This role of MSH and MSH-dependent enzymes as protectors against oxidative and acidic stress was also confirmed in *M. smegmatis* (34, 35). Moreover, *M. smegmatis* deficient in MSH showed a lower survival and higher sensitivity to hydrogen peroxide (36). This study is the first to examine the direct role of the MSH-recycling enzyme, Mtr, on the survival of *Mab in vitro* and *in vivo*, and after exposure to oxidative stress. To obtain these results, a novel *Mab mtr* knockout mutant, *Mab*∆*mtr,* was generated. The findings presented in this paper illustrate that *mtr* plays a role in the proliferation of *Mab* during macrophage and *G. mellonella* infection in which *Mab* missing *mtr* lacked the ability to proliferate inside both macrophages or larvae (Fig. 5 and 6b). Surprisingly, unlike MSH-deficient *M. smegmatis* being more sensitive to hydrogen peroxide (36), exposure of the *Mab*∆*mtr* mutant to hydrogen peroxide lowered the metabolic state of the bacteria but did not affect its viability (Fig. 4a and b).

Figure 1b demonstrated that one gene of the MSH biosynthesis, i.e., *mshB*, was upregulated when *mtr* was disabled in *Mab*. This may ensure the production of more MSH and indicate the possibility of a compensation mechanism within *Mab* to keep the internal MSH levels stable when the MSH recycling pathway is lost. On the other hand, other components might also play a role in a possible compensation mechanism. It was established by Ta et al. (37) that MshA-deficient *M. smegmatis* compensates for the loss of MSH by overexpressing an organic hydroperoxide resistance protein (Ohr) and ergothioneine (ESH) (13). However, we also illustrated that *Mab*∆*mtr* showed a decrease in the overall levels of the intracellular reduced thiol (Fig. 2). Since this test does not give us more information about the levels of each individual thiol, a compensation mechanism could still be present in some manner, which fails to fully compensate for the reduced MSH levels after knocking out *mtr*. Therefore, further research, including measurement of the internal MSH, ESH, and Ohr levels, is necessary to unravel the possibility of a compensation mechanism in the *Mab*∆*mtr* and to determine if *mshB*-upregulation is part of that compensation mechanism.

Another interesting discovery is the increased sensitivity of *Mab*∆*mtr* to the anti-mycobacterial drugs bedaquiline and moxifloxacin *in vitro* (Table 1). The sensitivity of the *Mab*∆*mtr* mutant to bedaquiline demonstrated a large shift, establishing a 5.1-fold reduction in IC50 and a 39.5-fold reduction in IC90 compared to WT *Mab*. This implies the possibility of a synergistic effect between Mtr-targeting drugs and bedaquiline. Bedaquiline, an inhibitor of the ATP-synthase, has been suggested as a potential drug for the treatment of *Mab* infections (38) and has been proven to rapidly deplete ATP in the bacteria (39). Furthermore, the ATP synthase is reported to be essential for mycobacteria, likely due to the essentiality of ATP (40). As a result, combining bedaquiline and a drug targeting Mtr during *Mab* infection could lead to an even higher depletion of bacterial ATP levels as a faster and greater reduction of ATP is perceived in the *Mab*∆*mtr* mutant when exposed to oxidative stress (Fig. 4a).

The same reason as for the higher sensitivity of *Mab*∆*mtr* to bedaquiline might be presented for moxifloxacin since it inhibits DNA replication by inhibiting DNA gyrase, an enzyme that works in an ATP-dependent manner (41). By inhibiting *mtr* as well as DNA gyrase, less ATP becomes available to DNA gyrase, which is already being mostly inhibited by moxifloxacin, hereby reinforcing the inhibition of the DNA replication.

As conclusion, our findings suggest that Mtr plays a role in the proliferation of *Mab* during infection and support the hypothesis of Mtr being a possible target for antimycobacterial drugs. These findings were possible as a result of the generation of a novel *Mab*∆*mtr* strain that will facilitate future research regarding the MSH biosynthesis and recycling pathway in *Mab*. Additionally, this paper suggests the potential activation of a partial compensation mechanism in *Mab* when the MSH recycling pathway is disabled. Finally, promising results were demonstrated regarding the use of a combined therapy including an anti-Mtr drug and bedaquiline, as *Mab* lacking *mtr* becomes more sensitive after exposure to bedaquiline *in vitro*.

## SUPPLEMENTAL MATERIAL

Supplemental data msphere.00669-23-s0001.docx Figures S1 and S2; Tables S1 and S2.

ASM does not own the copyrights to Supplemental Material that may be linked to, or accessed through, an article. The authors have granted ASM a non-exclusive, world-wide license to publish the Supplemental Material files. Please contact the corresponding author directly for reuse.
