# Supplementary material for: Mycothione reductase as a potential target in the fight against Mycobacterium abscessus infections
Source: mSphere. 2023 Dec 12;9(1):e00669-23. doi: 10.1128/msphere.00669-23 (PMC10826361; doi:10.1128/msphere.00669-23)
Supplement: Supplemental data — Figures S1 and S2; Tables S1 and S2. [file msphere.00669-23-s0001.docx]

**Supplemental Material**

**Table S1. Primers and oligo used for the construction and characterization of the *Mab*∆Mtr mutant.**


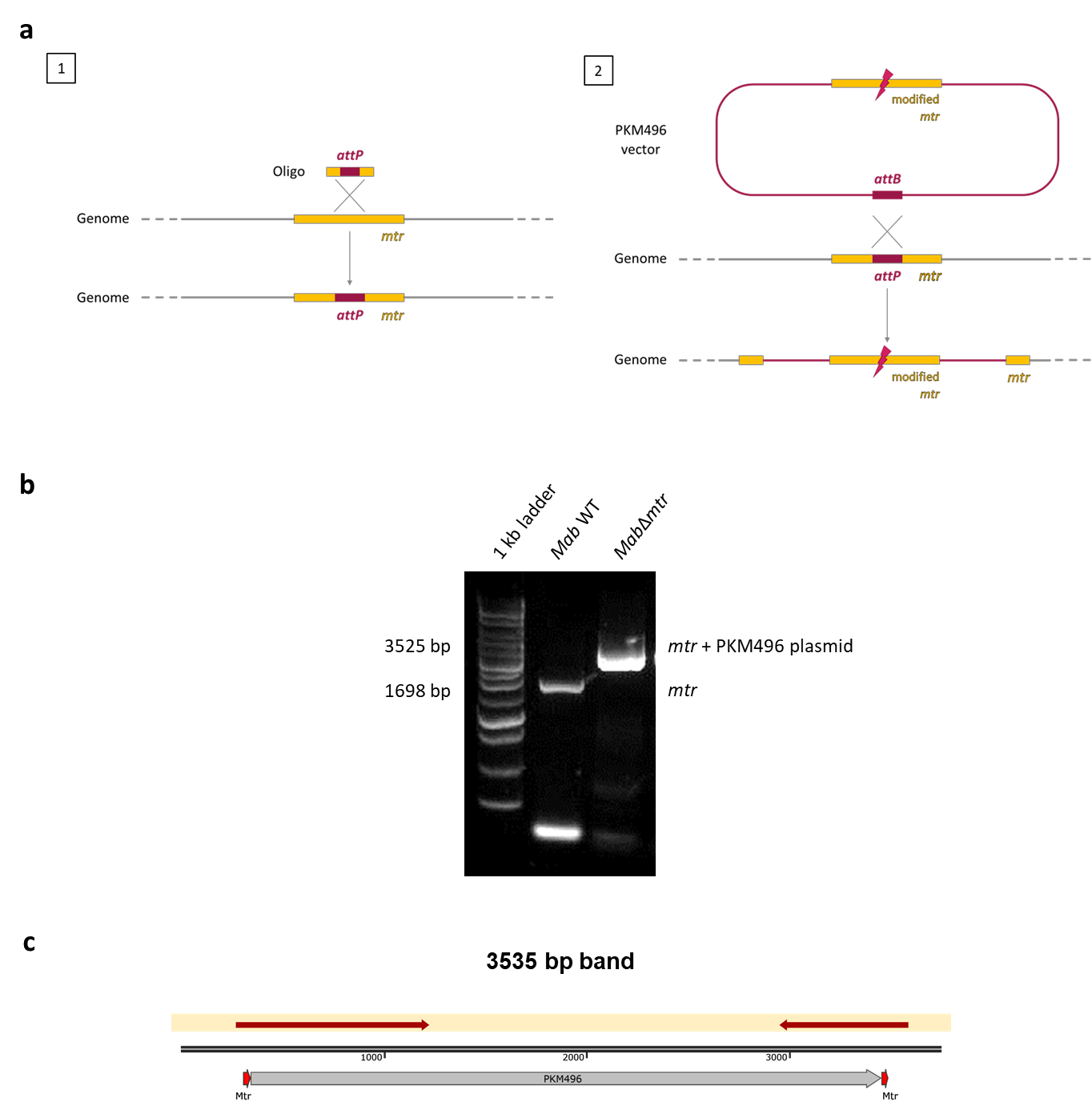


**Figure S1. Successful implementation of the ORBIT system to create a *Mab*∆*mtr* mutant.** To create *Mab*∆*mtr*, the bacteria were transformed with a total of two plasmids, PKM444 and PKM496, and an oligo. After transformation, the colonies were investigated by PCR and Sanger sequencing using primers to amplify the gene of interest. *Mab*∆*mtr* was successfully created when the PKM496 was incorporated in the middle of the gene of interest. **a)** Overview of the ORBIT system. Integration of the PKM496 containing a modified *mtr* is performed in a two-step process. First, an attP site is incorporated via the oligo in the gene of interest. Next, site-specific recombination occurs between the attP-containing genome and the attB-containing PKM496 vector, hereby interrupting the WT *mtr* gene. **b)** The PCR result showed a 1698 bp band generated for the WT while a 3525 bp band was generated for the tested colony, indicating that *mtr* was interrupted by the PKM496 plasmid in this colony. **c)** The generation of a *Mab*∆*mtr* mutant was confirmed by the Sanger sequencing results in which the sequence of the 3535 bp band perfectly aligned to a reference sequence including *mtr* interrupted by the PKM496 plasmid. Perfect alignment is illustrated by a filled, red-colored arrow.

**Table S2. Overview of the additional mutations detected using WGS in *Mab*∆*mtr* after transformation.** As expected with every transformation, a few base pairs substitutions were detected in *Mab*∆*mtr*, however, the biological relevance of these substitutions is not clear. The WT strain was used as reference.

**
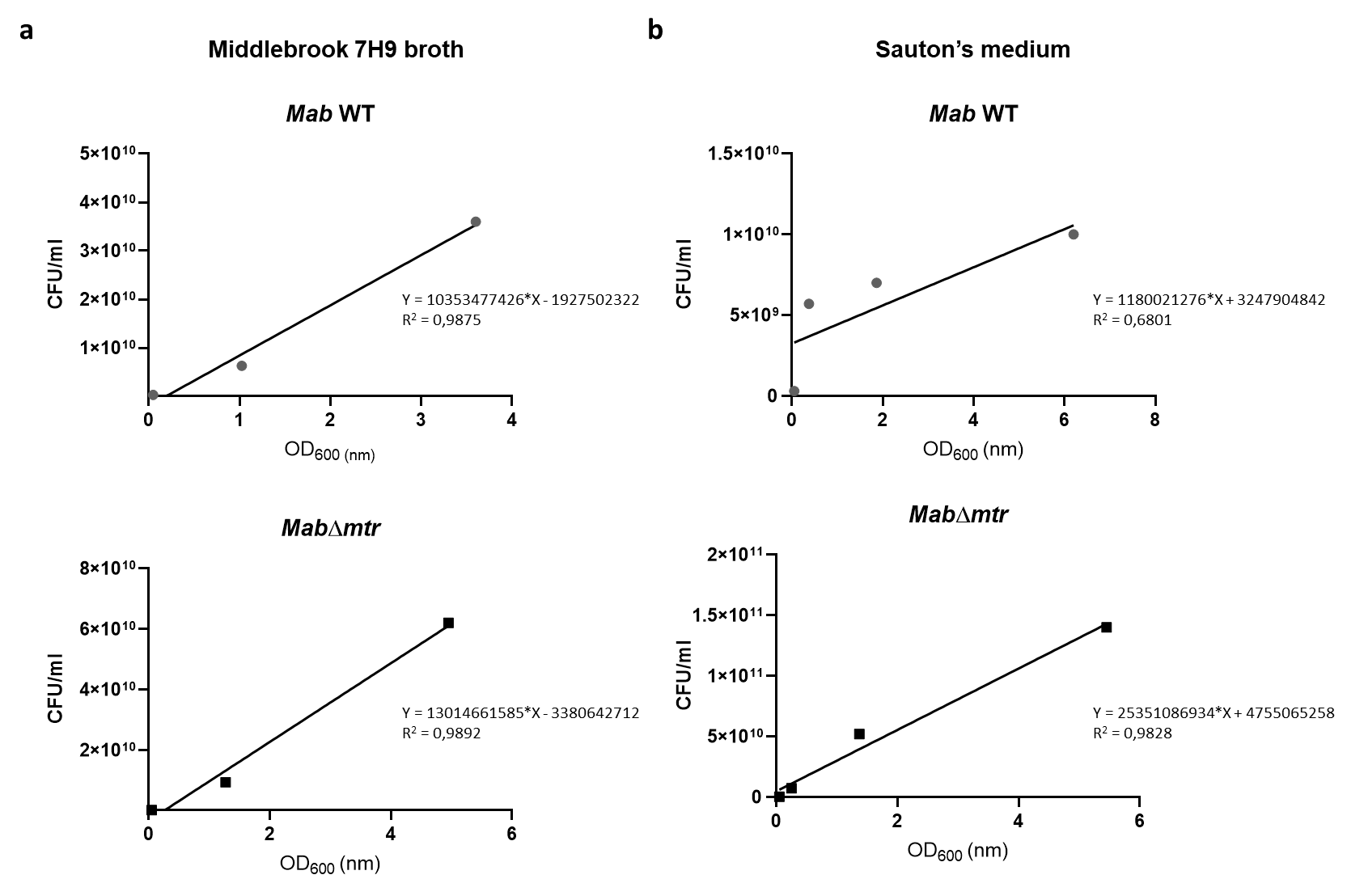
**

**Figure S2. *Mab*∆*mtr* mutant reaches higher plateau phase than *Mab* WT in a nutrient-poor medium.** Measurement of the CFU-OD_600_ proportion of the WT and *Mab*∆*mtr* mutant in Middlebrook 7H9 broth supplemented with 10% ADS, 0.2% glycerol and 0.05% tyloxapol (**a**) and nutrient-poor medium Sauton supplemented with 2% glycerol and 0.05% tyloxapol (**b**) shaking at 37°C. Before incubation, both strains were diluted to an OD_600_ of 0.05 in the corresponding medium. **a)** The CFU-OD_600_ proportion of *Mab* in 7H9 broth was not altered after knocking out *mtr*. **b)** In Sauton’s medium, however, a contrasting CFU-OD_600_ proportion is observed between WT *Mab* and *Mab*∆*mtr* in which *Mab*∆*mtr* reached a higher CFU count at a certain OD_600_. This confirmed the previously analyzed results of figure 3. Results are a representative of three independent experiments. A linear-regression was used to analyze the curves.
